# Book Review: Roma Minority Youth Across Cultural Contexts: Taking a Positive Approach to Research, Policy, and Practice

**DOI:** 10.3389/fpsyg.2021.752008

**Published:** 2021-10-06

**Authors:** Ana Kozina

**Affiliations:** Educational Research Institute, Center for Evaluation Studies, Ljubljana, Slovenia

**Keywords:** Roma (gypsies), youth, Positive Youth Development, minority—majority, context

The book *Roma minority youth across cultural contexts: Taking a positive approach to research, policy, and practice* centers research attention around widely underrepresented large ethnic minority group, that is the Roma minority. It uses and innovative combination of the three pillars of change: research, policy and practice. These are framed around the strength-based approach of Positive Youth Development Perspective. The book distinguishes itself from current edited books on Positive Youth Development by focusing on the Roma ethnic minority (one of the most marginalized and oppressed minority groups in Europe) and on strengths and resources for their optimal well-being. An ambitious and a rich overview of the complexities of Roma life in a variety of cultural settings is provided by the international, multidisciplinary and multi-sectorial experts' contributors.

The book is structured in three complex and intertwined parts: part 1—*The Current Situation of Roma*; part 2—*Theories on Roma Adaptation and Well-Being* and part 3—*Empirical Findings on PYD and Well-Being of Roma*. The added value of the book lies in (i) an increase in knowledge about this rapidly growing and highly stigmatized and disadvantaged population; (ii) centering around strength-based approach as opposed to deficit model so frequently applied in relation to Roma youth; (iii) inclusion of studies that focus individually or comparatively across major countries hosting this minority. Latter provides the opportunity both for a uniquely deep analysis of the Roma in a wide range of cultural contexts and draws inferences about commonalities and differences in the experience of Roma across cultures and nations.

In the first part, part 1—*The Current Situation of Roma*, it focuses on the current situation of Roma and their cultural context. A brief historical overview of major socio-demographic, cultural and contextual characteristics of Roma populations across different countries are presented in the Introduction. Chapter 1 (*The Roma Context*) adds historical outline as well as a summary of major socio-demographic, cultural and contextual characteristics of Roma. In addition, it compares these characteristics across different countries hosting Roma populations and their potential importance for Roma children and youth. Chapter 2 (*How Positive Youth Development Can Support Low-Income Roma Youth Living in the United States*) adds new perspective of the specifics of Roma living in the United States with centering around mechanisms that enables them to flourish. This first section concludes with a policy and programs overview focusing on policy and program development pursued for Roma youth in various countries as well as evidence on good practices and initiatives for Roma (e.g., Romani Early Years Network) in Chapter 3 (*Engaging Vulnerable Romani Youth in Provision of Early Childhood Services*).

Part 2—*Theories on Roma Adaptation and Well-Being*, presents theoretical background all the way from the traditional frameworks to the strengths-based Positive Youth Development model with a strong and particular emphasis on Roma youth. Chapter 4 (*Actualizing Change with Roma Youth and Their Communities: Theoretical and Conceptual Considerations*) focuses on current definition and operationalization of Positive Youth Development in the fields of human development and applied developmental science. It ends with a practical consideration of how existing, beneficial youth development interventions designed for youth with other ethnic minority backgrounds might be effectively adapted to improve the lives of Roma youth. A critical state-of-the-science overview of the literature on Roma youth and their developmental trajectories and outcomes are outlined in Chapter 5 (*Roma Youth: Positive Development and Challenges*). This second section largely illustrates how the Positive Youth Development approach can be through descriptive and intervention research applied to ethnic minority groups (non-Roma and Roma). For instance, specific positive youth development perspective actions required to be accompanied by more general multiculturalism policies aimed at creating the conditions for promoting positive development globally, even in the most stigmatized youth, such as Roma.

In the part 3 (*Empirical Findings on PYD and Well-Being of Roma*) consisting of 6 chapters, the book collects empirical findings on PYD and well-being of Roma youth across various contexts and countries. A unique and exciting overview on the well-being of Roma youth from several (in research literature mostly underrepresented) contexts is presented: in Chapter 6 (*Positive Youth Development: An Empirical Study of Roma Youth*), in Chapter 7 (*Reframing the Narrative: Revealing Positive Youth Development in the Self-Descriptions of Roma Adolescents*), in Chapter 8 (*Associations Between Social Connectedness and Academic Achievement Among Roma Youth in Eastern Europe*); in Chapter 9 (*Youth Development, Education, and Identity: A Case Study of Attitudes Toward Roma Youth in Hungary*); in Chapter 10 (*How Positive Youth Development Framework Can Improve Lives of Excluded People: The Case of Low-Income Roma Youth in Serbia*) and in Chapter 11 (*Out of the Margins: Reflections on Roma and Youth Development*). Here we can see a good example of how these contributions provide unique opportunity to apply the Positive Youth Development framework (primarily United States based) in the quest for generalized across different context, e.g., European, and across different socio-cultural settings, e.g., Roma youth. With its empirical evidence the book unfolds all the varieties of positive development of Roma youth with inclusion of mixed-methods approach and developing concussions based on the voices of youth. Finally, in their concluding chapter leading scholars integrate all content of the book by discussing key implications and future outlook. The concluding chapter (*Roma Youth Development in Context: What Next*) centers around policy implications and existing mismatch between Roma needs and governments inclusion efforts are discussed to provide practitioners with suggestions on improving life conditions of the next generation of Roma in a global perspective. They conclude with an innovative mixture of Positive Youth Development and multiculturalism to work “hand in hand” in providing systemic context for the support of positive development of Roma youth.

In my opining the book is “a must read” not only for the researchers, even though it provides a massive amount of strong empirical evidence of the varieties of contexts as well as possible strength-based interventions for Roma youth, but primarily to practitioners and policy makers as drivers of positive change. Policy and practice together can provide an excellent starting point as a combination of top and bottom-up approaches. The change of course starts in every individual in every family in every classroom in every context but it needs to be at the same time fostered and supported by the policy systems at national and EU level. This book presents an important step in the direction of improvement of the life conditions and perspectives of Roma youth.

## Author Contributions

The author confirms being the sole contributor of this work and has approved it for publication.

## Funding

This work was funded by ARRS (the Slovenian Research Agency; grant number: J5-1781).

## Conflict of Interest

The author declares that the research was conducted in the absence of any commercial or financial relationships that could be construed as a potential conflict of interest.

## Publisher's Note

All claims expressed in this article are solely those of the authors and do not necessarily represent those of their affiliated organizations, or those of the publisher, the editors and the reviewers. Any product that may be evaluated in this article, or claim that may be made by its manufacturer, is not guaranteed or endorsed by the publisher.

